# Retest reliability of measuring hip extensor muscle strength in different testing positions in young people with cerebral palsy

**DOI:** 10.1186/1471-2431-11-42

**Published:** 2011-05-25

**Authors:** Kate M Dyball, Nicholas F Taylor, Karen J Dodd

**Affiliations:** 1 School of Physiotherapy and Musculoskeletal Research Centre, La Trobe University, Bundoora, 3086. Australia

## Abstract

**Background:**

In young people with spastic diplegic cerebral palsy weakness of the hip extensor muscles are associated with limitations in activity. It is important that clinicians can reliably measure hip extensor muscle strength to monitor changes over time and the effects of any interventions. Previous research has demonstrated high reliability for measuring strength of all muscles of the lower limb, with the exception of the hip extensors. Therefore the aim of this study was to examine the retest reliability of measuring hip extensor strength in young people with cerebral palsy.

**Methods:**

Using a test-retest reliability research design, 19 participants with spastic diplegic cerebral palsy (Gross Motor Function Classification System Levels II and III) (mean 19 y 2 mo [S D 2 y 5 mo]) attended two testing sessions held 12 weeks apart. Three trials with a hand-held dynamometer were taken at each testing session in supine, prone and standing. Retest reliability was calculated with Intraclass Correlation Coefficients (ICC_(2,1)_) and with units of measurement (kilograms) converted to a percentage strength change.

**Results:**

ICC values ranged from .74 to .78 in supine, .75 to .80 in prone, and .73 to .75 in standing. To be 95% confident that real change had occurred, an individual's strength would need to increase 55 to 60% in supine, 86 to 102% in prone, and 102 to 105% in standing. To be 95% confident that real change had occurred across groups, strength would need to increase 4 to 8% in supine, 22 to 31% in prone, and 32% to 34% in standing. Higher ICC values were observed when three trials were used for testing.

**Conclusions:**

The supine testing position was more reliable than the prone or standing testing positions. It is possible to measure hip extensor strength with sufficient reliability to be able monitor change within groups using the supine position provided three trials are used during testing. However, there is insufficient reliability to monitor changes in hip extensor strength in individuals with cerebral palsy unless they exhibit very large strength increases.

## Background

A strong relationship has been demonstrated between lower limb muscle weakness and limitation of activity in young people with cerebral palsy [1-7]. The hip extensors, in particular, are important for many common every day functional activities such as being able to move from sit to stand, to climb steps and stairs, and to maintain upright posture during walking [8]. Hip extensor muscle weakness is one of the factors that can contribute to a gait pattern characterized by increased hip and knee flexion during stance, commonly described as crouch gait [9]. Crouch gait posture often develops and progresses during the adolescent growth spurt [10], due to growth adversely affecting weight to strength ratios and the development of joint malalignments [10]. With progression of crouch gait, anti-gravity support muscles including the hip extensors must work at a progressively greater proportion of their moment-generating capacity to maintain upright posture [11], resulting in gait deterioration, and increased dependence on gait aids when walking [9].

Due to the negative impacts of muscle weakness on function, progressive resistance strength training has become more common in young people with cerebral palsy [12-14]. This has led researchers and clinicians to become increasingly interested in evaluating the effects of interventions that aim to increase lower limb muscle strength. To monitor changes over time and to quantify changes in strength after intervention and be confident that the results are due to genuine changes in strength rather than measurement error, it is important to determine the reliability of muscle strength testing over clinically relevant periods of time. If interested in monitoring changes over time the most appropriate form of reliability to evaluate is retest reliability. A lack of reliability in measuring hip extensor muscle strength in young people with cerebral palsy may explain the equivocal results of progressive resistance strength training interventions on hip extensor muscle strength [15,16].

Previous studies investigating the retest reliability of testing the strength of lower limb muscles in young people with cerebral palsy, have reported high indices of retest reliability for hip flexors, hip abductors, knee flexors, knee extensors, ankle dorsiflexors and ankle plantarflexors [17,18]. However, the retest reliability of hip extensor strength has proven less reliable. Although Intraclass Correlation Coefficients ranging from .40 to .88 have been reported, retest reliability when calculated in terms of the units of measurement have been considered poor, with a large percentage of change in strength required to be considered a real change over and above measurement error. For example, an individual being tested in either of the two prone positions would have to improve their strength by more than 140%, and average increases in a group would need to exceed 54% for the change to be considered a real change with 95% confidence [17]. Changes of this magnitude are likely to be greater than those reported in most short-term progressive resistance training programs across a range of different health conditions, including cerebral palsy, where strength increases of about 25-30% are typically obtained [14]. A factor that may have contributed to the variable estimates of retest reliability for measuring hip extensors in young people with cerebral palsy is testing position. They have been tested in prone, with the hip in neutral [17,18] or in 45° hip flexion [19], and in supine with the hip in 90° flexion [18]. There is a need to investigate which testing position of the hip extensors in young people with cerebral palsy results in optimal retest reliability.

Another problem is that it remains uncertain how many and which trials should be used to represent the measure of muscle strength. There has been considerable variation in the number of trials and muscle force values used to calculate reliability in previous studies. For example, Taylor et al. [17] measured three trials and averaged the second and third trial for a measure of typical performance. In contrast, Crompton et al. [18] measured three trials, but used the peak force value from those three trials as the measure of strength. No previous studies have reported reliability calculated from only the first test trial, which could be relevant clinically, because testing with only one trial is quicker and so could be a more efficient way of measuring muscle strength.

Given these considerations, the aim of this research was to determine if hip extensor strength can be measured with sufficient retest reliability to monitor changes in strength of an individual and of a group of young people with cerebral palsy. For the purposes of this study, sufficient reliability required measurement error to be less than a 25% change in strength [14]. To achieve this aim we first needed to determine the optimal number of consecutive trials required to calculate measurement of hip extensor muscle strength and identify the most reliable position in which to assess the strength of the hip extensor muscles.

## Methods

### Participants

Using a test retest study design, participants were recruited from the wait-list control group of a randomised controlled trial. Therefore, the current study was conducted along-side a randomised controlled trial assessing whether a lower limb progressive resistance strength training program can improve the walking ability of young people with cerebral palsy. However, participants in the current study, who were in the control group in the larger study, did not receive any intervention between test and retest sessions of this reliability study. Participants were recruited through a state-based cerebral palsy register. From this register individuals who met the inclusion criteria were identified and contacted by letter informing them of the project and inviting them to contact the researchers if they were interested in participating. In addition, information flyers were handed out to potential participants attending the outpatients department of a large metropolitan tertiary children's hospital.

To be included volunteers needed to be aged 14 to 22 years and have spastic diplegic cerebral palsy, with a Gross Motor Function Classification System (GMFCS) level of II (youth walk in most settings without a mobility device) or III (youth walk using a hand-held mobility device) [20]. So they could cooperate with the testing procedures, volunteers also needed to be able to follow simple instructions. Volunteers were excluded if they had single event multi-level orthopaedic surgery within the previous two years, or if they had participated in a strength training program in the 6 months prior to the start of the trial. They were also excluded if they had contractures of more than 20° at the hip or knee as this would make it difficult for participants to assume the different positions for testing. Following a calculation used for determining appropriate sample sizes in retest reliability studies, and assuming an acceptable coefficient range of 0.7 to 0.9, the minimum sample size for this study was 19 participants [21].

### Procedure

The Human Ethics Committees of the children's hospital (HREC 2806) and the University (HEC 08-012) approved the trial, and written informed consent was obtained for each participant.

All testing was performed in a purpose built gait laboratory situated at a children's hospital. An accredited exercise physiologist experienced in the procedures of muscle strength testing and the use of a hand-held dynamometer measured and recorded the results of the strength testing. The tester worked in a gait laboratory at a Children's Hospital so was experienced in testing muscle function in young people with cerebral palsy. This tester was blinded to group allocation, therefore was unaware whether the participant was part of the experimental or control group of the larger randomised controlled trial. Muscle strength was measured with a hand-held dynamometer (Lafayette Instrument Company, Indiana, USA). Participants were asked to push as hard as they could, while the tester gradually increased force isometrically with the hand-held dynamometer over 3 seconds. This allowed the participant to adjust and to recruit the maximum number of muscle fibres. This test is known as a 'make' test, as distinct from a 'break' test where the tester attempts to overcome the participant's resistance [22]. Make tests have previously been shown to be more reliable than break tests and are therefore recommended for measuring muscle strength in children with cerebral palsy [23].

Each participant was tested in the three positions described in Table 1. The standing position has not been tested previously in young people with cerebral palsy but was chosen because the hip extensors are known to be important for gait and for the maintenance of upright stance, and therefore was a functionally relevant position to test. The prone position was modified from positions used in past studies where the hip was assessed from an extended position, by supporting it in 30° of flexion, so that the hip extensors were not contracting from their shortened range. The supine position was chosen because it tests the strength of the hip extensors when the person being tested is well stabilised. Three different positions were chosen to allow for comparison between positions. The left hip extensors were tested. Previous reliability studies of young people with spastic diplegic cerebral palsy reported no differences between testing the strength left and right lower limbs [17,18], and limiting the testing to one side limited any effects of fatigue and participant concentration during testing. For these reasons the left hip was chosen arbitrarily for testing.

**Table 1:** Testing positions

***Body Position***	***Joint Starting Position***	***Position of Resistance of HHD***
Supine	Hip flexed 90°, knee flexed at 90°	Posterior distal thigh
Prone	Hip flexed 30° and resting on a firm wedged surface, knee flexed 90°	Posterior distal thigh
Standing	Hip flexed 30°, knee extended	Posterior distal thigh

HHD, Hand-held dynamometer

At each test session, 3 trials were completed in each position, with a 90 second rest between each trial. The order of test position was randomly allocated using a random numbers table to minimize any series effects that fatigue may have had on the results. After completing the first test session, participants were instructed to continue with their typical daily activities. Participants were advised that they could attend physiotherapy in this time, providing it did not include a progressive resistance training program.

The second test session took place 12 weeks after the first session. No interventions that would be expected to change strength were implemented during the 12 weeks, so there was no expectation that muscle strength had changed between the first and second testing sessions. Also, the time between test and retest sessions should be clinically relevant, that is similar to the time over which a clinician would expect to monitor change if an intervention had been implemented. For our retest reliability study 12 weeks was a clinically relevant time over which to evaluate retest reliability of hip extensor muscle strength, because studies have shown that a period of six to twelve weeks is required to detect change in strength in young people with cerebral palsy after intervention [12]. The testing protocol used for session 2 was identical to that of session 1.

### Statistical analysis

Descriptive statistics, including means and standard deviations, were used to describe demographic data. Different combinations of the trials were used to calculate retest reliability: 1) the mean of all three trials, 2) the mean of trials one and two, 3) the mean of trials two and three, 4) the maximum measure from the three trials and, 5) the first trial only.

Retest reliability was measured in two ways, using a coefficient of reliability and units of measurement (kilograms). The coefficient of reliability, which can be referred to as relative reliability, was calculated using an Intraclass Correlation Coefficient (ICC_(2,1)_). Results below .60 represented poor reliability, .60 to .75 moderate reliability and values above .75 good reliability [24]. Reliability in terms of the units of measurement, which can be referred to absolute reliability, was calculated using 95% confidence intervals (CI). The 95% CI provides information about how much change would be required to be 95% confident that real change had occurred. The 95% CI was calculated for individual and group scores to illustrate the reliability of assessing muscle strength in an individual or in assessing strength of a group across two testing sessions. A 95% confidence derived from the difference between means of paired scores was calculated for the group score [15], according to the formula:

Where *Md *is the mean difference of retest minus test scores, *SDdiff *is the *SD *of the difference between retest and test scores, and *t_α_*is the value of t at which change is accepted with 95% confidence for a 2-tailed paired t-test and *N *is the number of participants. Ninety five percent CIs were also calculated for individual scores by substituting N = 1 into the equation [17]. These CIs which have also been termed the "limits of agreement" [25], are useful for clinicians, because they provide information about how much an individual would need to change to be 95% confident that real change had occurred. The 95% CIs were then converted into a percentage change required for the measurement to be greater than error. This was calculated by dividing the upper band CI for kilograms change by the original test score, and multiplying this value by 100.

## Results

Table 2 summarises the participants' demographic data. Nineteen participants were involved in the current study, nine males and ten females, with ages ranging from 14 years 9 months to 22 years 8 months of age. The level of disability for 12 of the participants was classified as GMFCS level II, with the disability level of the remaining 7 participants classified as GMFCS level III.

**Table 2:** Participant demographics

Sex (n)	Male	9
	Female	10
Age (y.mo)	Mean (SD)	19y2mo (2y5mo)
	Range	14y9mo to 22y8mo
Weight (kg)	Mean (SD)	60.1 (12.7)
	Range	40.7 to 83.5
Height (cm)	Mean (SD)	163.3 (8.8)
	Range	150.6 to 184.0
Ankle-foot orthoses (n)	Solid	4
	AFO not specified	2
	Hinged	2
	None	11
GMFCS Level (n)	II	12
	III	7
Gait Aid (n)	Single-point sticks	5
	Elbow crutches	1
	Kaye walker	1
	None	12

n, number of participants; y.mo, years and months; kg, kilograms; cm, centimetres; SD, standard deviation; GMFCS, Gross motor function classification system.

Results were variable when only the first trial or the mean of trials one and two were used to calculate reliability. For standing and supine the ICC values ranged from .55 to .64, indicating poor to moderate reliability, while for the prone position both ICC values of .83 indicated good reliability.

For relative reliability, ICC values calculated using the mean of all three trials, the mean of trials two and three and the maximum of the three trials were similar within and across positions (Table 3). ICC values ranged from .74 to .78 for measures taken in supine, .75 to .80 for measures taken in prone, and .73 to .75 for measures taken in standing. This indicates that all three positions had moderate to good reliability.

For absolute reliability, to be 95% confident that real change had occurred, an individual's strength would need to increase 8.6 to 9.3 kg (55 to 60%) in the supine position, 12.6 to 13.4 kg (86 to 102%) in the prone position, and 11.8 to 12.7 kg (102 to 105%) in the standing position. To be 95% confident that real change had occurred across groups, strength would need to increase 0.6 to 1.2 kg (4 to 8%) in supine, 3.1 to 3.7 kg (22 to 31%) in prone, and 3.7 to 3.9 (32% to 34%) in standing. These results were calculated using results for the mean of all three trials, the mean of trials two and three and the maximum.

**Table 3:** Retest reliability of measuring hip extensor muscle strength in different testing positions in young people with cerebral palsy (n = 19 left hips)

*Position*		*Mean test score (SD)*	*Mean retest score ± (SD)*	*ICC(2,1)*	*95% CI of ICC*		*Mean difference*	*SD difference*	*95% CI of unit of measurement*		*% change > error*	
					*Lower*	*Upper*			*Group mean*	*Individual*	*Group mean*	*Individual*
Standing	Trial 1	10.5 (5.6)	11.6 (6.3)	.64	.28	.84	1.1	5.07	-1.32 to 3.57	-9.53 to 11.78	34.9%	113.1%
	Mean (trials 1&2)	10.9 (5.8)	12.9 (8.2)	.63	.28	.84	2.0	5.92	-0.72 to 4.99	-10.31 to 14.58	46.8%	134.8%
	Mean (trials 2&3)	11.7 (6.5)	13.0 (7.9)	.75	.47	.89	1.3	5.09	-1.08 to 3.83	-9.33 to 12.07	33.0%	104.0%
	Mean (trials 1-3)	11.3 (6.2)	12.5 (7.3)	.72	.42	.88	1.2	5.00	-1.12 to 3.70	-9.23 to 11.80	33.6%	105.3%
	Maximum	12.6 (6.8)	13.9 (8.0)	.73	.43	.89	1.3	5.43	-1.28 to 3.95	-10.07 to 12.74	32.0%	101.5%
Prone	Trial 1	12.6 (7.4)	13.7 (8.7)	.83	.61	.93	1.0	4.70	-1.20 to 3.47	-8.74 to 11.01	28.3%	88.4%
	Mean (trials 1&2)	12.9 (7.5)	13.5 (8.9)	.83	.60	.93	0.6	4.95	-1.89 to 3.04	-9.83 to 10.98	24.2%	85.8%
	Mean (trials 2&3)	13.7 (8.0)	14.3 (8.8)	.75	.44	.90	0.6	6.08	-2.38 to 3.67	-12.13 to 13.42	27.6%	99.0%
	Mean (trials 1-3)	12.6 (8.1)	13.6 (8.9)	.79	.54	.91	1.0	5.56	-1.69 to 3.68	-10.69 to 12.69	30.6%	101.5%
	Maximum	14.6 (8.3)	14.7 (9.7)	.80	.53	.92	0.1	5.90	-2.82 to 3.05	-12.28 to 12.57	21.5%	86.3%
Supine	Trial 1	13.8 (6.7)	13.8 (6.9)	.55	.13	.80	0.0	6.52	-3.14 to 3.14	-13.70 to 13.70	23.6%	100%
	Mean (trials 1&2)	15.3 (8.2)	13.7 (6.5)	.62	.26	.83	-1.6	6.34	-4.71 to 1.44	-15.04 to 11.77	10.0%	77.2%
	Mean (trials 2&3)	15.7 (7.3)	13.9 (6.9)	.74	.45	.89	-1.8	4.94	-4.16 to 0.60	-12.15 to 8.59	4.3%	55.0%
	Mean (trials 1-3)	15.1 (6.7)	13.9 (6.8)	.75	.46	.89	-1.2	4.78	-3.46 to 1.15	-11.20 to 8.89	8.1%	59.4%
	Maximum	17.1 (7.7)	15.7 (7.9)	.78	.53	.91	-1.4	5.11	-3.87 to 1.05	-12.15 to 9.33	6.6%	54.8%

NB: Mean difference equals retest minus test strength score. Strength recorded in kilograms. SD, standard deviation; ICC, intraclass correlation coefficient; 95% CI of ICC, 95% confidence interval of ICC; 95% CI of units of measurement, 95% confidence interval of unit of measurement; % change > error, percentage of change required to be 95% confident the change is greater than error

## Discussion

The supine position was the most reliable of the three positions used in testing hip extensor muscle strength in young people with cerebral palsy because it demonstrated the smallest values of absolute reliability across the three testing positions. Although reliability indices (relative reliability) across the three testing positions appeared similar, the amount of change required to be 95% confident that real change over measurement error had occurred (absolute reliability) was less in the supine positions than the prone and standing test positions. For example strength increases of more than 8% across groups could be interpreted as true change when measured in the supine position; in contrast group increases of 31% and 34% would be required to be interpreted as true change when measured in the prone and standing positions, respectively. The supine position was stable for the participant and required participants to generate a force across gravity; these two factors may have enabled the participant to be able to generate a more consistent force isolated to the hip extensors, making the test more repeatable and, therefore more reliable.

The prone position was also stable for the participant but required them to generate a force against gravity. The need for participants to exert more effort in lifting the weight of their leg before exerting force on the dynamometer may have contributed to reduced reliability compared to the supine position. The standing position has not been evaluated before in the measurement of hip extensor strength in young people with cerebral palsy although high levels of retest reliability (ICC = .92) have been reported in using a modified standing position to assess hip extensor muscle strength in adults without impairment [26]. This position was thought to be advantageous because it is a more functional position to assess the strength of the hip extensors. However, in standing the participant must perform the dual task of maintaining the challenging testing position while performing the task. Dual tasking has been shown to make primary motor tasks, such as walking, more difficult in other neurological conditions [27]. The dual task may have made the performance of the test less consistent, and therefore reduced reliability.

The results suggest that measuring hip extensor strength in a group of young people with cerebral palsy can be measured with sufficient reliability in the positions of supine to monitor changes in strength. Measuring hip extensor strength in the supine position means that group changes of more than 8% could be confidently attributed to real change. Therefore, using hand-held dynamometry to quantify hip extensor strength is likely to be useful to clinicians and researchers who want to evaluate the effect of group interventions and programs to improve hip extensor strength with the aim of improving hip function during important every day functional activities such as walking.

Measuring changes in individuals is not as reliable as measuring changes across groups. For the supine position, percentage increases of 55% to 60% would be required to be 95% confident that real change had occurred. There are examples where strength training interventions in young people with cerebral palsy have led to improvements of this magnitude [2]. However, strength increases from interventions typically are of a lesser magnitude in the range of 25-30% [14]. Therefore, the results of the current study suggest that hip extensor strength is not able to be measured with sufficient reliability for clinicians to monitor typical changes for an individual prescribed a strength training program.

The results of the current study suggest that using the mean of all three trials, the mean of the second and third trials, or the maximum appears to have little impact on the calculation of reliability. However, when the first trial only, or the mean of the first and second trials was used, reliability was lower. For standing and supine, ICC values using the first trial only or the mean of the first two trials were below .64 (.55 to .64), indicating poor reliability. The results of this study, suggest that using the first trial only or the mean of the first and second trials is not as reliable as basing the estimate of strength on a combination of three trials. This is relevant clinically, because clinicians want to be able to test in the most reliable manner, but also the most efficient.

The results of our study also suggest that it might be misleading to rely only on coefficients, such as the ICC, to evaluate the reliability. Our results indicated little difference in the reliability coefficients between the three testing positions, all ranging from .73 to .80. However, clinicians and researchers are interested in whether observed change represents true change or measurement variability. This information is gained by expressing reliability in the units used for measurement. In terms of the units of measurement, our results indicated that the supine position for testing hip extensor muscle strength was more reliable than the prone or standing positions, since less change would be required to be interpreted as true change. Correlation coefficients do not indicate differences in repeated tests, rather the retest variability relative to the differences between subjects. For these reasons, it has been recommended that reliability be expressed in the units of measure and not only in terms of correlation coefficients [28].

This study has contributed to the literature by providing guidance about the most reliable method for measuring hip extensor strength in young people with cerebral palsy. The current study builds on previous research [17-19] by comparing three starting positions for testing, including the standing position, which had not been previously evaluated. The reliability coefficients in our study for testing in prone (.75 to .80) are comparable to those reported by van der Linden et al [19] (.75 to .83) but somewhat larger than values reported by Crompton et al [18] (.26 to .40). The reliability coefficients for testing in supine in our study (.74 to .78) are comparable to that reported by Crompton et al [18] (.79 to .82). Similar to Crompton, we concluded that the supine position to be more reliable than the prone testing position. The current study also adds to previous research by determining whether fewer than three trials can be used for testing, as has been used in previous trials [17-19].

### Limitations

However, there are some limitations. Only a subset of young people with spastic diplegic cerebral palsy and mild to moderate disability were evaluated. The criteria excluded young people with more severe and different types of cerebral palsy who may also benefit from monitoring muscle strength. The sample size of the current study was relatively small, although the number of participants was equal to the sample size estimated for a study of this nature [21]. A larger sample size may serve to narrow the confidence intervals about the reliability coefficient. Also, it needs to be considered how a static measurement of hip extensor muscle strength, as measured with a hand-held dynamometer relates to dynamic hip extensor muscle action during functional tasks such as walking and this could be the subject of further research. It could also be considered whether a retest interval of 12 weeks between measures was a limitation since many retest reliability studies use much shorter retest intervals. However, because the choice of retest interval should be related to the intended purpose of a measurement [29], and monitoring muscle strength in young people with cerebral palsy would involve the reassessment of muscle strength over 6 to 12 weeks [12], we think that the choice of a 12 week retest interval was appropriate. Finally, the results of the current study do not provide information about other forms of reliability, such as inter-tester reliability. We evaluated retest reliability as we felt it was the most clinically relevant for hip extensor muscle strength in young people with cerebral palsy where a clinician or researcher is interested in monitoring change over time. Despite this, research on inter-tester reliability, which evaluates the repeatability between two raters at one time has also demonstrated moderate to good levels of reliability (ICC ranged from .67 to .82) using the make test to measure hip extensor muscle strength in the supine position in young people with cerebral palsy [23].

## Conclusions

Strength testing in a supine position using a portable manual hand-held dynamometer appears to have sufficient reliability to measure group mean changes in hip extension strength in young people with cerebral palsy and is a more reliable testing position than prone or standing. The results of this study suggest that three trials should be used for testing, but it does not matter whether the maximum, or means of all three or the second and third trial are used to calculate reliability. Strength testing does not have sufficient reliability to monitor changes in hip extensor strength in individuals with cerebral palsy unless they exhibit very large strength increases.

## Competing interests

The authors declare that they have no competing interests.

## Authors' contributions

KMD participated in the design of the study, assisted with data collection and drafted the manuscript. NFT participated in the design of the study, led statistical analysis and helped draft the manuscript. KJD conceived of the study, participated in its design and helped draft the manuscript. All authors read and approved the final manuscript

## Pre-publication history

The pre-publication history for this paper can be accessed here:

http://www.biomedcentral.com/1471-2431/11/42/prepub
